# Impacts of Lesion Characteristics on Procedures and Outcomes of Chronic Total Occlusion Recanalization With Antegrade Guidewire True Lumen Tracking Techniques: A Substudy of Taiwan True Lumen Tracking Registry

**DOI:** 10.3389/fcvm.2022.769073

**Published:** 2022-03-01

**Authors:** Chi-Jen Chang, Shih-Chi Liu, Cheng-Ting Tsai, Jen-Fang Cheng, Chien-Lin Lee, Chia-Pin Lin, Chi-Hung Huang, Jun-Ting Liou, Yi-Chih Wang, Juey-Jen Hwang

**Affiliations:** ^1^Cardiovascular Division, Department of Internal Medicine, Chang Gung Memorial Hospital, Taoyuan, Taiwan; ^2^Cardiovascular Division, Department of Internal Medicine, Fu Jen Catholic University Hospital, New Taipei City, Taiwan; ^3^Cardiovascular Division, Department of Internal Medicine, MacKay Memorial Hospital, Taipei, Taiwan; ^4^Cardiovascular Division, Department of Internal Medicine, National Taiwan University Hospital, Taipei, Taiwan; ^5^Cardiovascular Division, Department of Internal Medicine, Far Eastern Memorial Hospital, New Taipei City, Taiwan; ^6^Cardiovascular Division, Department of Internal Medicine, Cathay General Hospital, Taipei, Taiwan; ^7^Cardiovascular Division, Department of Internal Medicine, Tri-Service General Hospital, Taipei, Taiwan

**Keywords:** chronic total occlusion, true lumen tracking, J-CTO score, long-term outcome, antegrade approach

## Abstract

**Background:**

Lesion characteristics were shown to predict procedural success and outcomes in chronic total occlusion (CTO) recanalization. However, diverse techniques involved in these studies might cause potential heterogeneity.

**Objective:**

The study aimed to test the impacts of lesion characteristics on CTO intervention with a pure antegrade wiring-based technique.

**Methods and Results:**

We studied consecutive 325 patients (64.5 ± 11.1 years, 285 men) with native CTO lesions intervened by a single operator with an antegrade-based technique between August 2014 and July 2020. Forty-seven patients with antegrade procedural failure (20 with pure antegrade wiring failure and 27 with back-up retrograde techniques) were compared to 278 patients with antegrade-only procedural success. With a median follow-up of 30.8 (16.1–48.6) months, 278 patients with procedural success were further assessed for target vessel failure (TVF: cardiac death, target vessel myocardial infarction [MI], and target lesion revascularization [TLR]). Patients with antegrade procedural success had a lower percentage of history with bypass graft (4 vs. 15%, *p* = 0.004) and lower Multicenter Chronic Total Occlusion Registry of Japan (J-CTO) score (2.1±1.3 vs. 3.4 ± 1.0, *p* < 0.001), when compared to those with antegrade failure. The J-CTO score was independently associated with procedural failure (odds ratio = 2.5, 95% CI = 1.8–3.4) in multivariate analysis. However, only clinical features, such as female gender (hazard ratio [HR] = 4.3, 95% CI = 1.4–13.1), estimated glomerular filtration rate <60 ml/min/1.73 m^2^ (HR = 3.2, 95% CI = 1.0–9.9), and old MI (HR = 4.5, 95% CI = 1.5–12.8), but not J-CTO score, could predict long-term TVF in multivariate Cox regression model.

**Conclusion:**

The feasibility of the antegrade guidewire-crossing technique for native CTO intervention was highly determined by lesion characteristics. With such a simpler technique, the prognostic impact of lesion complexity shown in studies with multiple recanalization techniques was negligible. This suggested antegrade true lumen tracking techniques deserved to be tried better even for CTO lesions with higher complexity.

## Introduction

In recent years, advances in recanalization devices and retrograde crossing techniques have improved the overall procedural success of the percutaneous coronary intervention (PCI) for chronic total occlusion (CTO) (1–3). Furthermore, emerging CTO-PCI algorithms help decision-making for an optimal CTO-PCI strategy before and during the whole procedure (4–6). Nevertheless, for CTO interventionists, especially those with fewer experiences or limited devices, pure antegrade wiring for true lumen tracking remains to be the more accessible and less invasive technique with lower risks among the whole CTO recanalization methods in routine practice. This procedure is particularly by far the majority in the field of CTO-PCI.

The Multicenter Chronic Total Occlusion Registry of Japan (J-CTO) score (7) was developed to grade the difficulty in crossing a CTO lesion within 30 min and to evaluate the overall success rate (8). Its utility of outcome prediction has also been validated in several subsequent CTO studies (9, 10). Theoretically, each of the five parameters included in the J-CTO score was basically more relevant to procedural difficulty for antegrade, but not for retrograde approaches. Furthermore, when evaluating long-term prognosticators, the impacts of the false lumen with variable sizes and lengths created by antegrade reentry devices or several retrograde techniques (11, 12) still remain unclear. However, either the J-CTO registry or other CTO studies evaluating the impact of lesion characteristics on procedures and outcomes enrolled a substantial proportion of patients (14.5–53.1%) recanalized with retrograde approaches (7, 9, 13, 14). The diverse percentage of reentry or retrograde techniques used in these studies could cause potential bias in investigating predictors of overall procedural success and long-term prognosis. To improve the limitations of multiple techniques involved in prior studies addressing the predictability of lesion characteristics for procedural success and long-term prognosis, the purpose of the study was to assess the performance of the J-CTO score for recanalization feasibility and predictors for outcomes with an antegrade wiring-based true lumen tracking and crossing technique in an independent, high-volume single-operator cohort.

## Methods

### Study Design and Population

**Figure 1:**
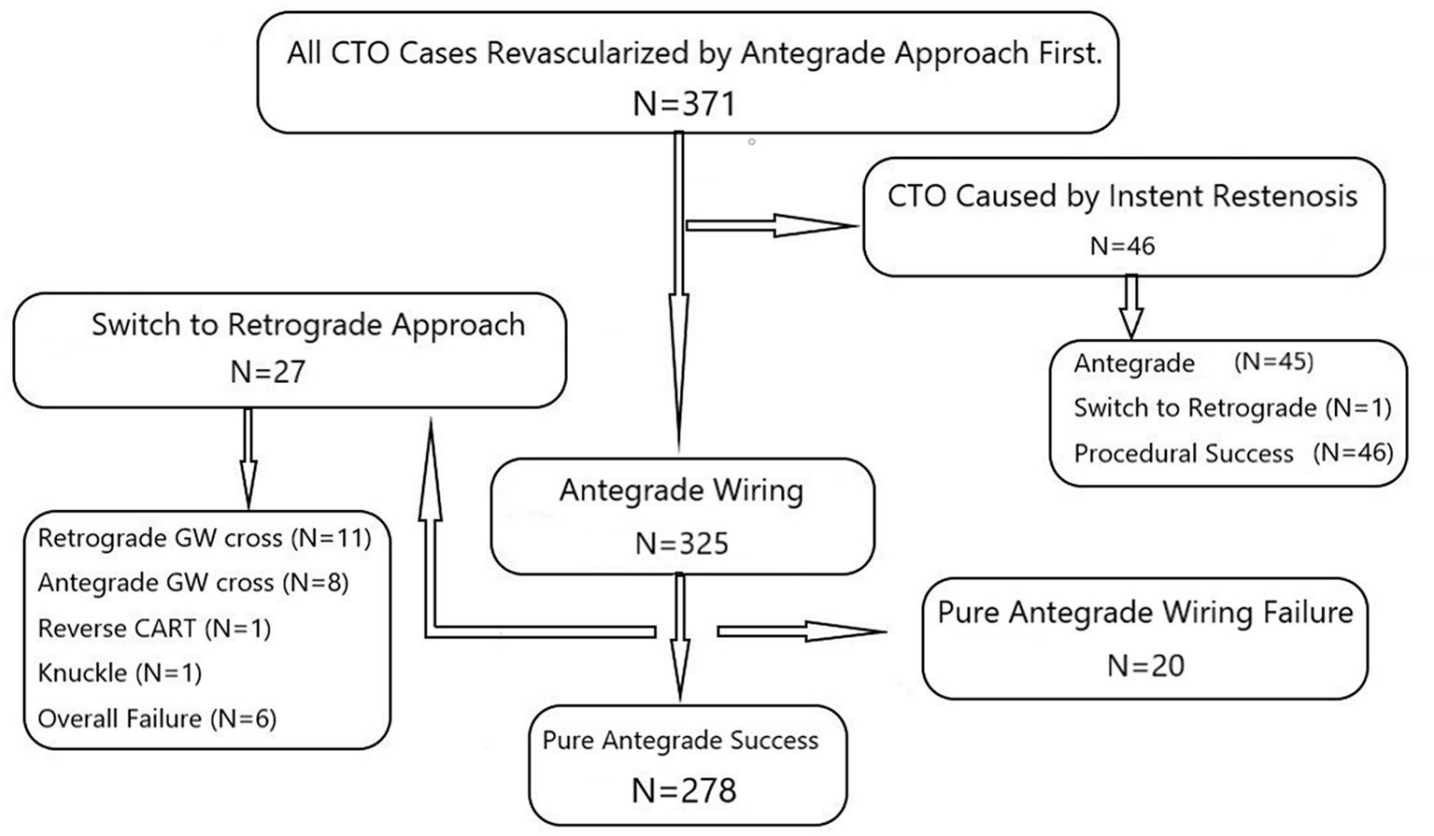
The single-operator cohort with the antegrade-first and wiring-based technique showed 325 procedures for native CTO cases. Twenty-seven cases switching to retrograde techniques and twenty cases with pure antegrade wiring failure were both regarded as the group with antegrade wiring failure (*N* = 47). CTO, chronic total occlusion; CART, controlled antegrade and retrograde tracking; GW, guidewire.

The study was a sub-study of the Taiwan True Lumen Tracking (TTLT) multicenter registry conducted by seven high-volume CTO operators in seven independent medical centers in north Taiwan. This is a prospective nonrandomized registry, and retrospective enrollment could be allowed in the individual center. Briefly, the criterion of successful “true lumen tracking” in the registry was no loss of any side-branch ≧ 1.5 mm by quantitative coronary angiography (QCA) throughout the entire CTO body, including 5 mm before and after the occluded segment in the final angiogram. If not fulfilled, the procedure was considered to be failed. Based on the purpose of the current study, data from the operator with all antegrade-first and the highest percentage (91.7%) of the pure antegrade approach used for all CTO cases among the seven centers were chosen for further analyses. The single-operator cohort included a total of 371 consecutive patients with CTO who underwent coronary revascularization in National Taiwan University Hospital between August 2014 and July 2020 (Figure 1). After exclusion of 46 cases with CTO due to instent restenosis, 325 patients (64.5 ± 11.1 years, 285 men) with native CTO lesions were enrolled. The study was conducted according to the Declaration of Helsinki and was approved by the Institutional Ethic Committee of National Taiwan University Hospital, and the written informed consents were obtained.

### Procedures

Angiographic multivessel disease was defined as stenoses ≥ 50% by QCA in at least two of the three major epicardial coronary arteries. The definition of coronary CTO was as follows: coronary lesions with thrombolysis in myocardial infarction (MI) grade 0 flow of at least 3-month duration. The duration was estimated from the occurrence of clinical events, such as MI in the target vessel territory, sudden onset or worsening of chest symptoms, or by angiography (15, 16). The J-CTO score counting was assessed by two independent operators (Dr. SC Liu and Dr. CL Lee). According to the consensus of the strategy in the TTLT registry, antegrade approach with guidewire-based true lumen tracking and crossing technique was always tried first irrespective of individual J-CTO parameter or the overall score. For all antegrade procedures, the soft guidewires were mainly Fielder series, such as FC, XT-A, and XT-R (Asahi Intecc Medical, Japan). Gaia series (Asahi Intecc Medical, Japan) guidewires were introduced in Taiwan in August 2015. The stiff guidewires included the CONFIANZA Pro Series (Asahi Intecc Medical, Japan), the ProVia Series (Medtronic Co., Minneapolis, MN, USA), and the Progress Series (Abbott Vascular, Santa Clara, CA, USA). During recanalization procedures, guidewires escalating or de-escalating, or the timing to switch to parallel wire technique or retrograde approach, was mainly determined by the tactile feedback and discretion of the operator. Each successful recanalization procedure was achieved by gentle and cautious manipulation of the guidewires, and intravascular ultrasound for intima or subintimal tracking was not used for the whole procedures included in the study. Any re-entry devices were not available in this period and thus not used in the study. In addition, intravascular imaging was strongly recommended but not mandatory in the registry, and the aid of intravascular imaging at any time point of the procedure was also left at the operator's discretion. For each intervention, the appropriate amount of reference air kerma was judged for the sake of whole procedure completion by the operator. With the optimized use of balloons, stents, or any plaque modifications devices, the success of CTO-PCI was defined as restoration of thrombolysis in MI grade 3 anterograde flow, postprocedural stenosis <30%, and no loss of any side-branch ≧ 1.5 mm throughout the CTO segment as the consensus of TTLT registry in the final assessment of angiography. No occurrence of in-hospital major adverse cardiac and/or cerebro-vascular events, such as cardiac death, Q-wave MI, stroke, or any repeat target lesion revascularization (TLR), was necessary for the definition of procedural success.

For evaluating factors that predict the feasibility of antegrade wiring, those with failed pure antegrade guidewire crossing (*N* = 20, Figure 1) and necessity of back-up retrograde techniques (*N* = 27) in recanalization procedures were both considered to be the group with antegrade procedural failure (total *N* = 47). They were compared to those with successful antegrade wiring (*N* = 278, 64.7 ± 11.2 years, 242 men).

All patients with successful procedures were required to receive 100 mg of aspirin daily lifelong in addition to clopidogrel (75 mg/daily) for a period following the standard of current guidelines.

### Follow-Up of Patients

The 278 patients with pure antegrade procedural success were further analyzed for long-term clinical outcomes. Clinical events were defined according to the Academic Research Consortium (ARC) definitions (17). Target vessel failure (TVF) included cardiac death, target vessel MI, and clinically driven percutaneous, or surgical TLR. TLR was defined as any restenosis (diameter stenosis ≥ 50%) from 5 mm distal to 5 mm proximal to the initial occluded segment. Stent thrombosis is defined as definite, probable, or possible according to the ARC criteria. Clinical data were collected by regular hospital visits or telephone contact if necessary. Angiographic follow-up was performed only when symptoms recurred with/without positive non-invasive stress tests or documented restenosis by coronary CT angiography.

### Statistical Analysis

Statistical analysis was performed using the R 3.5.3 software (R Foundation for Statistical Computing, Vienna, Austria). In statistical testing, a two-sided *p* ≤ 0.05 was considered statistically significant. The distributional properties of continuous variables were expressed by mean ± SD and categorical variables were presented by frequency and percentage. In addition, the survival curve for the time from the index procedure to follow-up was estimated by the Kaplan–Meier method. The subjects lost to follow-up were right-censored at the last follow-up dates. In univariate analysis, the unadjusted effect of each parameter was examined using Wilcoxon rank-sum test, chi-square test, and Fisher's exact test as appropriate for the data type. Next, multivariate analysis was conducted by logistic regression analysis to calculate odds ratio (OR) and 95% CI to select a set of independent predictors of wiring failure of antegrade CTO-PCI. Cox proportional hazards model estimated by hazard ratio (HR) and 95% CI was used to analyze the effect of risk factors on time to cardiac events in patients who had successful antegrade PCI for CTO.

## Results

### Comparisons of Clinical Features

For all native CTO lesions shown in Figure 1, the overall success rate of pure antegrade techniques with only guidewires for true lumen tracking and the crossing was 86% (278/325). The clinical characteristics of the whole patients and comparisons between those with successful and failed antegrade guidewire-crossing are described in Table 1. Patients with failed antegrade procedures only had a higher percentage of history with coronary artery bypass graft surgery than those with procedural success (15 vs. 4%, *p* = 0.004). The other clinical features were similar between the two groups.

**Table 1:** Comparisons of clinical features between successful and failed antegrade procedures.

	**All** **(*N* = 325)**	**Successful procedure** **(*N* = 278)**	**Failed procedure** **(*N* = 47)**	***P*-value** **Success vs. failure**
Age (years)	64.5 ± 11.1	64.7 ± 11.2	63.5 ± 10.0	0.489
Male (%)	285 (88%)	242 (87%)	43 (91%)	0.393
Hypertension (%)	247 (76%)	215 (77%)	32 (68%)	0.171
Diabetes (%)	122 (38%)	103 (37%)	19 (40%)	0.660
Dyslipidemia (%)	227 (70%)	194 (70%)	33 (70%)	0.953
Smoking (%)	115 (35%)	100 (36%)	15 (32%)	0.592
Old MI (%)	70 (22%)	62 (22%)	8 (17%)	0.417
CABG (%)	19 (6%)	12 (4%)	7 (15%)	0.004
CHF (%)	60 (18%)	50 (18%)	10 (19%)	0.896
eGFR < 60 ml/min/1.73 m^2^ (%)	79 (24%)	72 (26%)	7 (15%)	0.104
Stroke (%)	16 (5%)	14 (5%)	2 (4%)	0.992

*CKD, chronic kidney disease; CHF, congestive heart failure; CABG, coronary artery bypass graft; eGFR, estimated glomerular filtration rate; MI, myocardial infarction*.

### Comparisons of Angiographic and Procedural Characteristics

**Figure 2:**
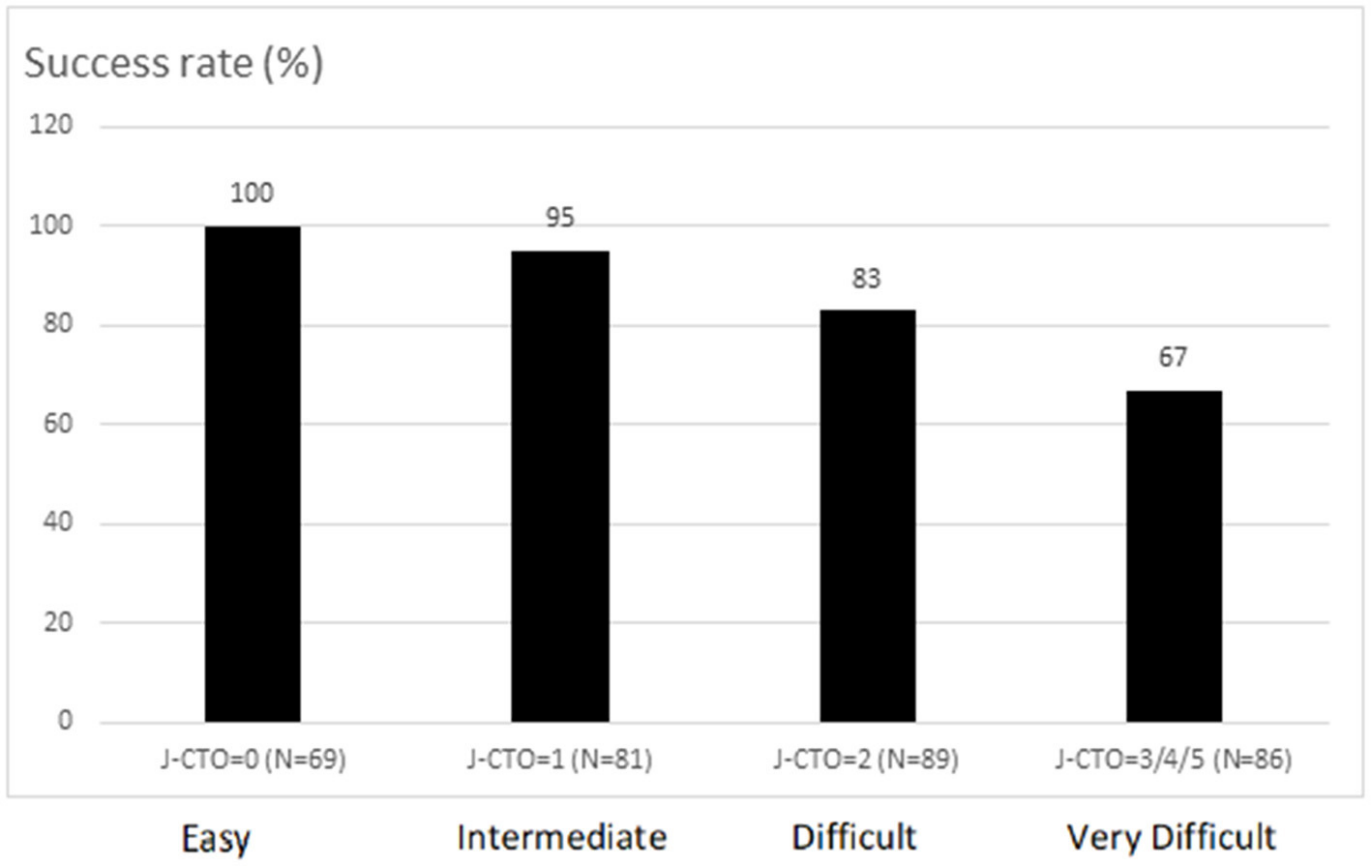
The success rate of pure antegrade wiring-based chronic total occlusion (CTO) recanalization for each category according to Multicenter Chronic Total Occlusion Registry of Japan (J-CTO) score.

Among the whole 325 patients, 298 patients (92%) had multivessel disease (Table 2). There were borderline lower percentage of right coronary artery (RCA) CTO (42 vs. 57%, *p* = 0.05) and higher percentage of transradial-only catheterization (73 vs. 49%, *p* = 0.001) in patients with successful procedures. The parallel wire technique was used in 42 (13%) procedures. It was significantly less used in the group with antegrade success than that with antegrade failure (8 vs. 40%, *p* < 0.001). Regarding lesion characteristics, the J-CTO score was much higher in patients with failed antegrade procedures (3.4 ± 1.0 vs. 2.1 ± 1.3, *p* < 0.001). The success rate of pure antegrade recanalization for each category of J-CTO score is shown in Figure 2. The presence of individual parameters for counting the J-CTO score in the failed group, except for the presence of calcification, was also significantly greater than that in the successful group. The application of intravascular imaging in the study was higher for those with successful antegrade wiring (50 vs. 30%, *p* = 0.012). Notably, there remained 30% of imaging tools used in the failed group, because some patients (*N* = 21) in this group still had final procedural success with the aid of retrograde techniques (Figure 1). Further, the amount of contrast volume (165 ± 74 vs. 244 ± 102 ml, *p* < 0.001) and the fluoroscopy time (50 ± 128 vs. 109 ± 32, *p* < 0.001) were both significantly lower in patients with successful antegrade wiring. Among the whole successful antegrade procedures, stenting, with the majority (246/253, 97%) of drug-eluting stent (DES), was performed in 91% of all lesions. The average number, size, and total length of stents used for each CTO lesion were 1.8 ± 0.9, 2.7 ± 0.3, and 55.3 ± 27.0 mm, respectively.

**Table 2:** Comparisons of angiographic and procedural characteristics between successful and failed antegrade procedures.

	**All** **(*N* = 325)**	**Successful procedure** **(*N* = 278)**	**Failed procedure** **(*N* = 47)**	***P*-value** **Success vs. Failure**
Multivessel dz. *(N)*	298 (92%)	258 (93%)	40 (85%)	0.060
**Lesion Location** ***(N)***				0.127
LAD *(N)*	118 (36%)	104 (37%)	14 (30%)	0.316
LCX *(N)*	62 (19%)	56 (20%)	6 (13%)	0.235
RCA *(N)*	144 (44%)	117 (42%)	27 (57%)	0.050
LM *(N)*	1 (0.3%)	1 (0.4%)	0	0.682
Transradial *(N)*	227 (70%)	204 (73%)	23 (49%)	0.001
Transfemoral *(N)*	60 (18%)	47 (17%)	13 (28%)	0.079
Radial+Femoral *(N)*	38 (12%)	27 (10%)	11 (23%)	0.007
J-CTO score	2.2 ± 1.4	2.1 ± 1.3	3.4 ± 1.0	<0.001
J-CTO ≧ 2	224 (69%)	179 (64%)	45 (96%)	<0.001
J-CTO ≧ 3	140 (43%)	101 (36%)	39 (83%)	<0.001
Blunt stump *(N)*	175 (54%)	136 (49%)	39 (83%)	<0.001
Occlusion ≧ 20 mm *(N)*	157 (48%)	119 (43%)	38 (81%)	<0.001
Occluded length (mm)	22.4 ± 9.9	20.6 ± 8.5	32.6 ± 11.1	<0.001
Bending *(N)*	185 (57%)	144 (52%)	41 (87%)	<0.001
Calcification *(N)*	178 (55%)	147 (53%)	31 (66%)	0.096
Retry case *(N)*	35 (11%)	23 (8%)	12 (26%)	<0.001
Intravascular Imaging *(N)*	152 (47%)	138 (50%)	14 (30%)	0.012
IVUS *(N)*	146 (45%)	132 (47%)	14 (30%)	
OCT *(N)*	6 (2%)	6 (2%)	0	
Parallel wire *(N)*	42 (13%)	19 (8%)	23 (40%)	<0.001
Multivessel PCI *(N)*	136 (42%)	122 (44%)	14 (30%)	0.070
Contrast volume (ml)	177 ± 84	165 ± 74	244 ± 102	<0.001
RAK (mGy)	6,234 ± 4,655	5,576 ± 4,028	10,319 ± 6,088	<0.001
DAP (μGym^2^)	40,104 ± 30,297	35,549 ± 25,874	68,911 ± 39,584	<0.001
Fluoroscopy time (min)	59 ± 35	50 ± 28	109 ± 32	<0.001
Drug-eluting stent *(N)*		246 (88%)		NA
BMS *(N)*		7 (3%)		
Balloon or DCB *(N)*		25 (9%)		
Average stent No. *(N)*		1.8 ± 0.9		NA
Stent size (mm)		2.7 ± 0.3		NA
Stent length (mm)		55.3 ± 27.0		NA

*BMS, bare-metal stent; DAP, dose area product; DCB, drug-coated balloon; IVUS, intravascular ultrasound; LAD, left anterior descending; LCX, left circumflex; LM, left main; NA, non-applicable; PCI, percutaneous coronary intervention; RAK, reference air kerma; RCA, right coronary artery; OCT, optical coherence tomography*.

### Multivariate Analysis for Predictors of Pure Antegrade Wiring Success

After adjusting age, gender, history of bypass graft surgery, all other clinical features listed in Table 1, presence of multivessel disease, and RCA-CTO, the J-CTO score remained the only predictor of antegrade wiring failure (OR = 2.5, 95% CI: 1.8–3.4, *p* < 0.001; Table 3). Regarding the individual parameter of J-CTO score, each but the presence of calcification is also independently associated with antegrade wiring failure as shown in Table 3.

**Table 3:** Multivariate logistic regression analysis for independent factors associated with overall antegrade procedural failure.

	**Adjusted odds ratio (95% CI)**	***p*-value**
J-CTO	2.5 (1.8–3.4)	<0.001
Blunt stump	5.6 (2.5–12.7)	<0.001
Occlusion ≧ 20 mm	5.2 (2.4–11.4)	<0.001
Bending > 45 degrees	6.9 (2.8–17.1)	<0.001
Retry case	3.7 (1.6–8.2)	0.002

### Kaplan–Meier Curves for Long-Term Outcomes

**Figure 3:**
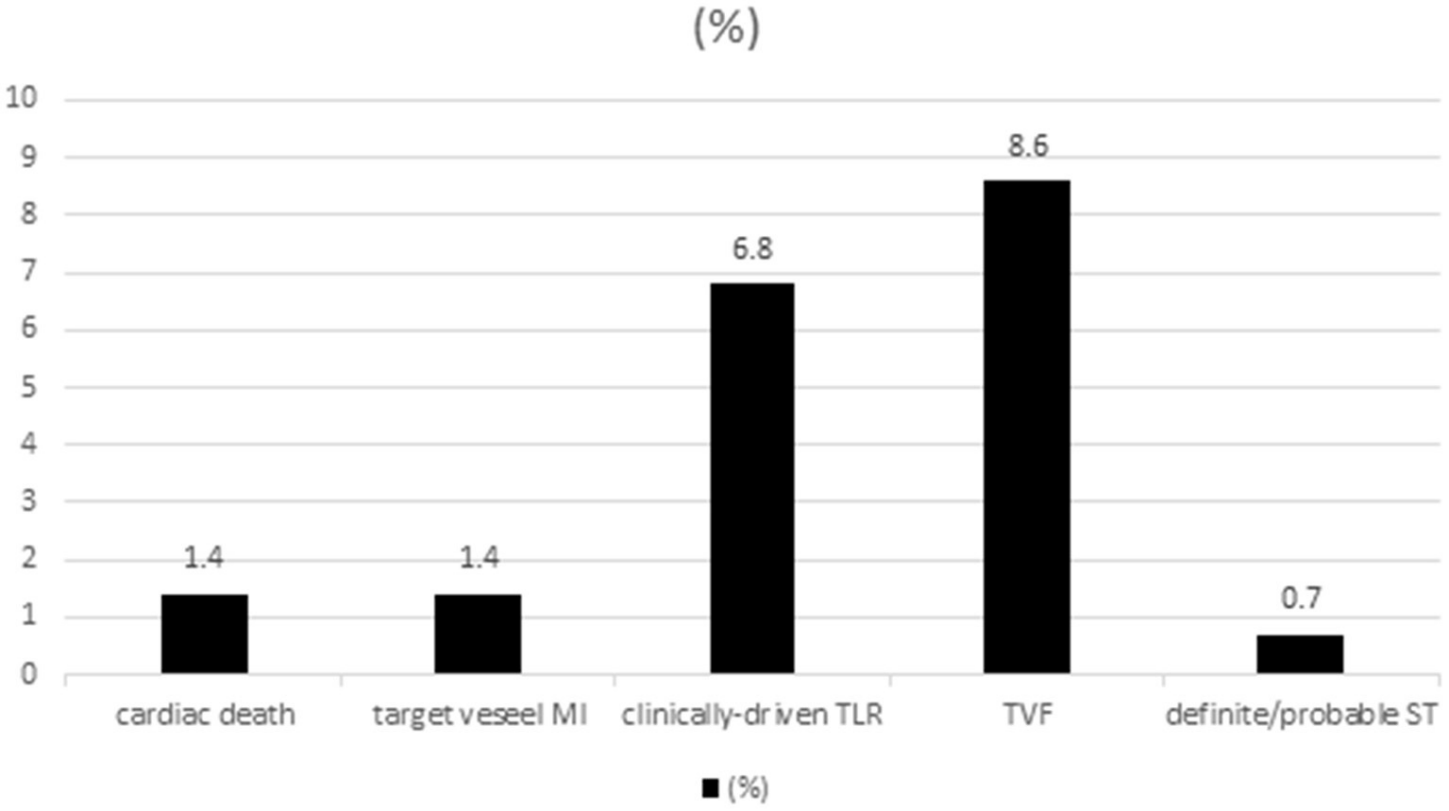
Long-term outcomes of the 278 patients with successful chronic total occlusion- percutaneous coronary intervention (CTO-PCI) by pure antegrade wiring techniques.

**Figure 4:**
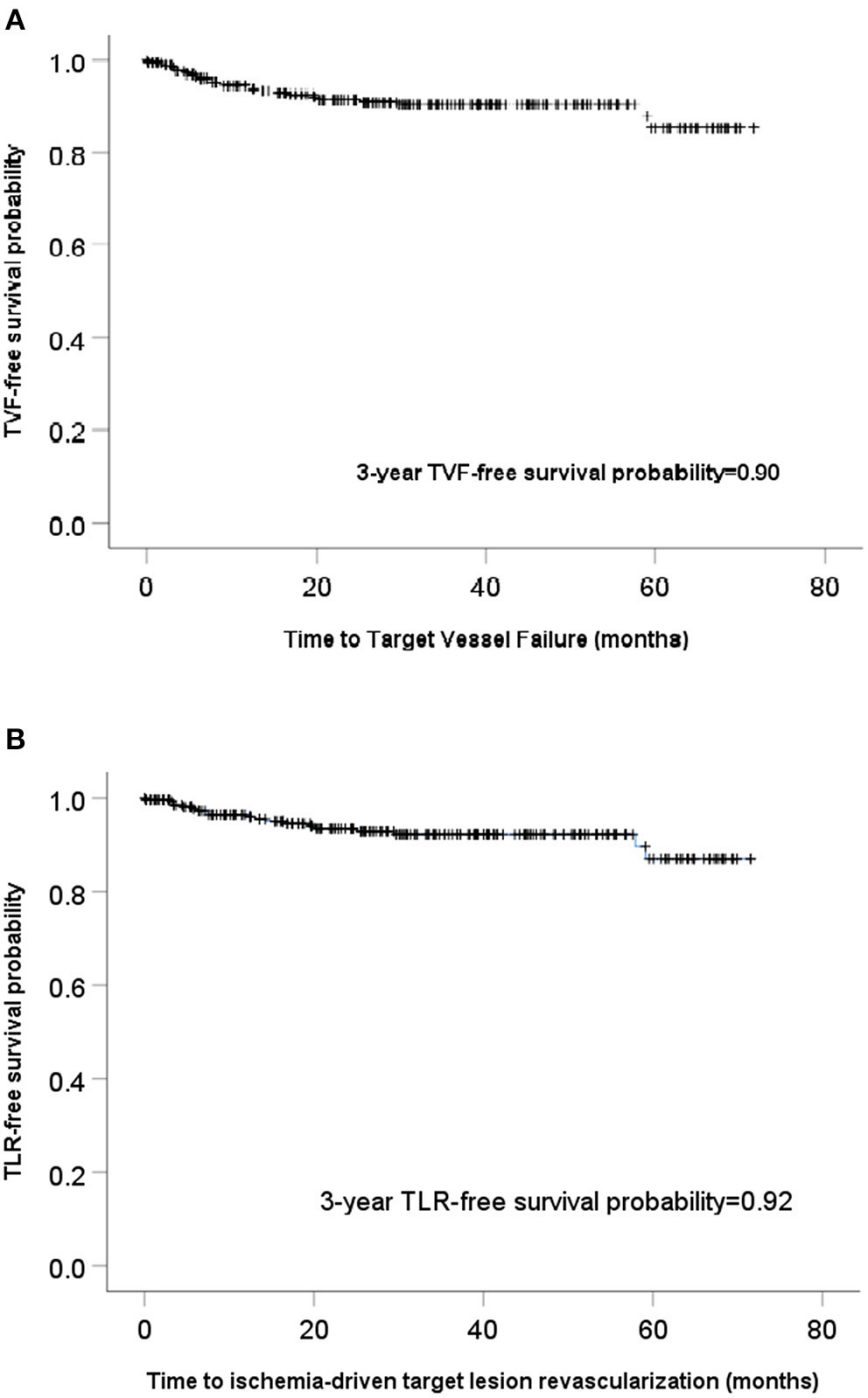
Kaplan–Meier curves showed the overall target vessel failure (TVF) **(A)** and clinically-driven target lesion revascularization (TLR)-free survival **(B)**.

With a median follow-up of 30.8 (16.1–48.6) months, the long-term outcomes of the whole patients with successful pure antegrade guidewire-crossing and recanalization for CTO are shown in Figure 3. The Kaplan–Meier curve showed that the overall TVF- and clinically driven TLR-free survival probability at 3 years were 0.90 (Figure 4A) and 0.92 (Figure 4B), respectively.

### Multivariate Analysis for Predictors of Long-Term TLR- and TVF-Free Survival

The J-CTO score was relevant neither to TVF (*p* = 0.248) nor to TLR (*p* = 0.154) in univariate analysis. After adjusting age, gender, and possible factors shown in univariate analysis, such as the history of bypass graft surgery, multivessel PCI at index procedure, and use of intravascular imaging, long-term TVF was independently associated with female gender (HR: 4.3, 95% CI = 1.4–13.1, *p* = 0.009), old MI (HR: 4.5, 95% CI = 1.5–12.8, *p* = 0.006), and estimated glomerular filtration rate (eGFR) < 60 ml/min/1.73 m^2^ (HR: 3.2, 95% CI = 1.0–9.9, *p* = 0.045). Ischemia-driven TLR was independently associated with female gender (HR: 5.1, 95% CI = 1.4–18.4, *p* = 0.015), and old MI (HR: 3.8, 95% CI = 1.2–12.7, *p* = 0.028; Table 4).

**Table 4:** Results of multivariate cox proportional hazard models for factors associated with long-term TLR and TVF.

**Multivariate analyses**	**Adjusted hazard ratio (95% CI)**	***p*-value**
**TLR**		
Female	5.1 (1.4–18.4)	0.015
Old MI	3.8 (1.2–12.7)	0.028
**TVF**		
Female	4.3 (1.4–13.1)	0.009
Old MI	4.5 (1.5–12.8)	0.006
eGFR < 60 ml/min/1.73 m^2^	3.2 (1.0–9.9)	0.045

*CKD, chronic kidney disease; MI, myocardial infarction; TVF, target vessel failure; TLR, target lesion revascularization*.

## Discussion

The registry study showed the feasibility of antegrade wiring-based true-lumen tracking techniques for all-comer CTO lesions. It reassured the applicability of lesion characteristics as J-CTO score to predict a procedural success of CTO recanalization in such a manner. Unlike previous CTO studies demonstrating the J-CTO score as a prognosticator, long-term outcomes of those with successful antegrade recanalization were only relevant to clinical features in the current study. This suggested that the possible prognostic contribution from false lumen creation by multiple techniques for complex lesions could be negligible if true lumen tracking and the crossing were cautiously done by antegrade wiring techniques only.

Besides the introduction of J-CTO score for predicting successful guidewire crossing within 30 min and grading the difficulty of CTO intervention (7, 8), several scoring systems, such as Prospective Global Registry for the Study of Chronic Total Occlusion Intervention (PROGRESS) CTO score, CT Registry of Chronic Total Occlusion Revascularization (CT-RECTOR) score, and Clinical and Lesion (CL)-related score, have also been shown to be associated with final angiographic success (18–20). The majority of the parameters derived from angiography in these scoring systems were basically relevant to the difficulty of antegrade procedures, and only few were directly linked to the feasibility of retrograde techniques. This is one of the reasons that the retrograde approach is favored in those with a higher score of the J-CTO or other grading systems. However, several studies to validate the J-CTO score (13, 14, 21) or to compare it with another scoring system (9, 22, 23) inevitably enrolled a substantial proportion of CTO lesions recanalized by retrograde techniques (14.5–53.1%). In addition, the presence of instent CTO (9, 21) and the use of antegrade dissection and reentry devices (21) were also found in some studies. Although all studies confirmed the impacts of lesion characteristics on procedural success, the underlying heterogeneity regarding lesions, techniques, and devices could partially compromise the predictability of these scoring systems. Therefore, the current study included a single-operator and antegrade-first cohort with a high preponderance (298/325 = 92%) of pure antegrade guidewire tracking and crossing techniques for native CTO lesions. Those switched to retrograde techniques were also considered as the antegrade wiring failure group even final procedural success was achieved. The study, with a comparable success rate to that in studies with variable techniques, reconfirmed the crucial role of lesion characteristics as predictors of procedural feasibility for an antegrade wiring-based manner.

Regarding the five parameters for calculating the overall J-CTO score, each was also shown to be independently associated with antegrade procedural failure, except for the presence of calcification in the study. In the J-CTO registry, calcification within the CTO segment, regardless of the severity, was considered as an independent marker of guidewire crossing within 30 min with either antegrade or retrograde procedures. However, its association with overall technical failure was unanswered. On the other hand, only severe, but not mild or moderate, calcification included in the CL-related score has been shown to predict successful first attempt PCI for CTO (20). Moreover, the efficiency of moderate-severe calcification to predict procedural success was even not demonstrated in the PROGRESS CTO study (18). For CTO interventionists, the presence of calcification would suggest a tight lesion with a longer duration. However, its distribution sometimes could serve as a roadmap to ensure that the guidewire was close to the true lumen, particularly, when the collaterals were not filling the target vessel well or the guidance of the retrograde guidewire was absent. This advantage at least partially neutralized the detrimental impacts of calcification on the antegrade-based technique for CTO recanalization as shown in the study.

The association between long-term prognosis and lesion characteristics as the J-CTO score has also been documented in several studies (9, 10). However, only clinical features, such as gender, old MI, and impaired renal function, could serve as prognosticators in the present study. In fact, diverse devices and techniques with different percentages involved in these registries could potentially cause confounding results in evaluating prognosticators after successful CTO-PCI. Fundamentally, more retrograde techniques or reentry devices were necessary for cases with more complex lesions as a higher J-CTO score (13, 14). Thus, the possibility of subintimal or false lumen creation must be higher due to multiple techniques used than pure antegrade wiring for true lumen tracking. Since the extent of subintimal is difficult to be quantified, its impacts on long-term outcomes remained to be determined. Imaging by intravascular ultrasound, Finn et al. (24) showed a higher unadjusted rate of 1-year TVF in patients with subintimal tracking than those with intraplaque tracking, and the presence of subintimal tracking was shown to be correlated with a high J-CTO score. Another study investigating the contribution of the retrograde approach on the outcome of CTO-PCI also showed that 4-year TVF increased with the application of the retrograde approach, mostly due to re-occlusion or TLR (25). Based on the correlation between the higher lesion complexity and the more use of retrograde, or dissection and reentry techniques recommended by current algorithms for CTO-PCI, the possible false lumen with variable sizes or lengths created might play a role in the worse outcomes for these patients. In clinical practice, it is nearly not possible for an antegrade guidewire to go back to the distal true lumen successfully if a substantial gap between the guidewires and occluded intima existed, or a large and long false lumen was created during wiring. Therefore, successful antegrade guidewire crossing with sizable side-branch preservation could ensure, if any, a relatively limited subintimal space created within the CTO segment. This could probably be accompanied by negligible lesion- or technique-dependent impacts on long-term prognosis as shown in the study.

Nevertheless, the retrograde approach as the backup procedure remained promising in the study. For overall 325 CTO procedures, the success rate could be increased from 86% (278/325) by pure antegrade wiring techniques to 92% (299/325) by the aid of retrograde techniques (Figure 1). Further, the success rate of difficult (83%) and very difficult lesions (67%) recanalized by pure antegrade wiring techniques was lower than the average (86%) (Figure 2). This suggested that an earlier switch from the antegrade-first strategy to hybrid techniques in the presence of interventional collaterals could be workable in lesions with J-CTO ≧ 2 if any trouble was encountered after reasonable attempts of antegrade wiring.

### Study Limitations

There were limitations of the study. First, this was a single-operator cohort study. Since the procedural familiarity of CTO intervention was different from operator to operator, the study results could not be generalized for all CTO interventionists. Nonetheless, the study investigating a simple CTO recanalization strategy minimized the inevitable heterogeneity and possible inconsistent results in multicenter registries. Second, intravascular imaging was used in 50% of patients with successful antegrade intervention. The judgment of true lumen tracking and the crossing was partly based upon the fundamental assumption of antegrade wire-crossing discussed above, side-branch preservation, and operator's experiences and therefore could not be completely confirmed. However, the definition of procedural success, such as preservation of all significant side branches, was rather strict, when compared to the prior studies with diverse recanalization techniques. Third, the overall procedure time was not recorded. The antegrade guidewire-tracking technique was considered to be time-consuming, especially for lesions with higher complexity. In addition, the percentage of multivessel and complete revascularization at the index procedure was high (42%, Table 2) due to the regulation of the health insurance system and patients' preferences. Both situations would possibly contribute to a much longer procedure time and higher radiation. Nevertheless, the overall fluoroscopy time and total radiation dose listed in Table 2 could still serve as comparisons with other CTO studies.

## Conclusions

The single-operator cohort data showed the feasibility of antegrade-first and pure wiring techniques for all-comer CTO lesions. Angiographic lesion characteristics as the J-CTO score were still shown to be highly relevant to procedural success in such kind of intervention. Moreover, with such a simpler technique to recanalize CTO lesions, the prognostic impact of lesion complexity shown in prior studies involving multiple recanalization techniques could be negligible. It suggested that the antegrade wiring with true-lumen tracking technique deserved to be tried better even for CTO lesions with higher complexity.

## Data Availability Statement

The raw data supporting the conclusions of this article will be made available by the authors, without undue reservation.

## Ethics Statement

The studies involving human participants were reviewed and approved by National Taiwan University Hospital. The patients/participants provided their written informed consent to participate in this study.

## Author Contributions

Y-CW: drafted the manuscript and contributed the data. All authors were involved in critically revising the manuscript and designed the study.

## Funding

This study was supported grant from the Prof. De-Lu Wu's Medical Foundation, and Taiwan Health Foundation, Taipei, Taiwan.

## Conflict of Interest

The authors declare that the research was conducted in the absence of any commercial or financial relationships that could be construed as a potential conflict of interest.

## Publisher's Note

All claims expressed in this article are solely those of the authors and do not necessarily represent those of their affiliated organizations, or those of the publisher, the editors and the reviewers. Any product that may be evaluated in this article, or claim that may be made by its manufacturer, is not guaranteed or endorsed by the publisher.
